# DECANT: decoupling mechanism from context in single-cell drug perturbation representation

**DOI:** 10.1093/bioinformatics/btag662

**Published:** 2026-09-07

**Authors:** Ren Qi, Wenjie Teng, Xin Yang, Yue Cheng, Alexey K Shaytan, Bin Liu

**Affiliations:** School of Computer Science and Technology, Beijing Institute of Technology, Beijing 100081, China; School of Computer Science and Technology, Beijing Institute of Technology, Beijing 100081, China; School of Computer Science and Technology, Beijing Institute of Technology, Beijing 100081, China; Zhongguancun Academy, Beijing 100094, China; School of Artificial Intelligence, Jilin University, Jilin 130012, China; Department of Biology, Lomonosov Moscow State University, Moscow 119991, Russia; School of Computer Science and Technology, Beijing Institute of Technology, Beijing 100081, China; Zhongguancun Academy, Beijing 100094, China; SMBU-MSU-BIT Joint Laboratory on Bioinformatics and Engineering Biology, Shenzhen MSU-BIT University, Shenzhen 518172, China

## Abstract

**Motivation:**

Single-cell chemical perturbation profiling offers a powerful opportunity to organize drugs by shared mechanism-associated transcriptional responses, but observed transcriptional responses are entangled with contextual variation from cell identity, dose, and treatment time. As a result, models that perform well in perturbation-response prediction may still learn latent spaces dominated by context-associated structure rather than transferable drug-associated signal. We developed DECANT to learn mechanism-aligned perturbation representations that remain stable across context shifts while preserving response fidelity.

**Results:**

DECANT represents each perturbation as a matched treated–control cell set and separates a context-suppressed, mechanism-aligned perturbation representation from context-dependent response information. The resulting mechanism-aligned perturbation space is shaped to support drug-level retrieval and biological interpretation. Under a fixed drug-level unseen-compound benchmark, DECANT achieved the strongest overall response-difference profile among adapted published perturbation models and strong pseudo-bulk baselines across gene- and program-level metrics. Beyond prediction, DECANT produced embeddings that remained stable across changes in dose, cell line, and treatment time, recovered drug neighborhoods enriched for shared mechanism-family annotations, and linked these neighborhoods to interpretable downstream consequence programs. Ablation analyses showed that mechanism–context decoupling provided the main signal-separation backbone, whereas retrieval-oriented shaping was critical for organizing local representation-space geometry. These results support DECANT as a framework for learning context-robust, mechanism-aligned perturbation representations from single-cell transcriptional responses, providing a basis for mechanism-aligned perturbation analysis and representation-based compound prioritization.

**Availability and implementation:**

The DECANT web server is publicly available at http://bliulab.net/DECANT. All source code and analysis scripts are available at https://github.com/bliulab/DECANT and archived on Zenodo at https://doi.org/10.5281/zenodo.21216567.

## 1 Introduction

Large-scale single-cell chemical perturbation profiling is changing how drug action can be studied (McFarland *et al.* 2020, Srivatsan *et al.* 2020). By measuring transcriptional responses at cellular resolution across compounds, doses, and biological contexts, these datasets provide an opportunity not only to predict perturbed expression states, but also to identify shared mechanism-associated response programs among compounds and to characterize response structures that remain stable across changing experimental and biological conditions (Lamb *et al.* 2006, Subramanian *et al.* 2017). This distinction is important because the central question in chemical biology and translational pharmacology is rarely limited to whether a compound changes gene expression. Broader AI applications in drug response prediction, combination therapy, repositioning, and molecular design further highlight the need for mechanism-aware compound comparison (Zeng *et al.* 2022, Zhang *et al.* 2024, Qi *et al.* 2025, Ren *et al.* 2025). A more informative objective is to determine whether related compounds converge onto context-robust shared response programs that support comparison of compounds not observed during training (Rees *et al.* 2016).

A major obstacle is that single-cell drug responses are inherently entangled. Observed transcriptional profiles reflect both drug-associated signal and context-associated variation from cell identity, dose, treatment time, and state-dependent response heterogeneity (Luecken and Theis 2019, Stuart *et al.* 2019). The same compound can therefore induce quantitatively different responses across contexts, whereas different compounds assayed in the same context can inherit substantial similarity from shared cellular background or response magnitude (Barretina *et al.* 2012, Iorio *et al.* 2016). As a result, accurate perturbation-response prediction does not necessarily imply that the learned latent space is organized by transferable drug-associated structure. In this setting, the key challenge is not simply perturbation prediction, but learning mechanism-aligned perturbation representations under context entanglement.

Recent computational methods have substantially advanced the modeling of single-cell perturbation data. Generative approaches such as scGen helped establish perturbation prediction as a tractable learning problem (Lotfollahi *et al.* 2019), while factorized models such as BioLord introduced more explicit representations of biological variation and out-of-distribution inference (Piran *et al.* 2024). Chemistry-aware and compositional frameworks, including chemCPA, further extended perturbation modeling by incorporating molecular information (Hetzel *et al.* 2022), and related architectures such as PRnet and PerturbNet have improved the ability of neural models to predict or transfer drug-induced responses (Qi *et al.* 2024,  Qiao *et al*. 2025b, Yu *et al.* 2025). These methods provide an important foundation for the field (Liang *et al.* 2023, Liang *et al.* 2024, Chen *et al.* 2025, Qiao *et al*. 2025a, Xie *et al.* 2025, Zhang *et al.* 2025). However, most existing frameworks are primarily optimized to reconstruct, infer or extrapolate expression responses. They are not explicitly designed to learn a drug-level representation whose geometry is stable across context shifts, supports retrieval of biologically related compounds, and remains interpretable in terms of downstream biological programs.

This limitation becomes particularly important under fixed drug-level unseen-compound evaluation, a setting related to the unseen-drug evaluation problems considered in recent single-cell perturbation-response models (Hetzel *et al.* 2022, Qi *et al.* 2024, Yu *et al.* 2025). In this setting, all measurements from a held-out compound are excluded from training, and the model must generalize to drugs that were not observed during model fitting. The scientific question is therefore not only whether plausible responses can be predicted for held-out compounds, but also whether the resulting representation captures drug-associated structure rather than context-entangled similarity. Consequently, improvement over weak neural baselines alone is insufficient evidence for a useful perturbation representation. A stronger framework should preserve drug-associated signal across biological and experimental shifts, suppress context-associated confounding in the mechanism representation, and organize local neighborhoods to support biological interpretation.

Here we present DECANT, a framework for learning context-robust, mechanism-aligned perturbation representations from matched treated, and control single-cell profiles. DECANT represents each perturbation as a matched treated–control cell set and separates a context-suppressed drug-associated latent representation from context-dependent response information. The context-suppressed representation is optimized to preserve drug-associated perturbational signal that remains stable across cell line, dose, and treatment time, whereas context-dependent residual information is retained for accurate gene- and program-level response prediction. In parallel, the drug-level space is shaped for retrieval and biological interpretation against independent mechanism-family annotations.

We evaluated DECANT under a fixed drug-level unseen-compound protocol against adapted published perturbation models and strong pseudo-bulk or bag-level baselines. DECANT achieved the strongest overall benchmark profile across gene- and program-level response metrics, and ablation analyses identified mechanism–context decoupling and retrieval-oriented shaping as the main contributors to response fidelity and local representation-space organization.

Beyond benchmark performance, DECANT produced a representation space with properties relevant to *post hoc* mechanism-aligned analysis. Same-drug embeddings remained stable across changes in dose, cell line and treatment time, whereas different drugs measured in the same context remained well separated. At the drug-prototype level, local neighborhoods were enriched for canonical mechanism-family annotations, linked to interpretable downstream consequence programs, and retained family-specific structure after controlling for overall response magnitude. Together, these results support DECANT as a framework for moving beyond context-entangled perturbation prediction toward context-robust, mechanism-aligned perturbation representation learning from single-cell chemical perturbation data.

## 2 Methods

### 2.1 Overview of the DECANT framework

**Figure 1:**
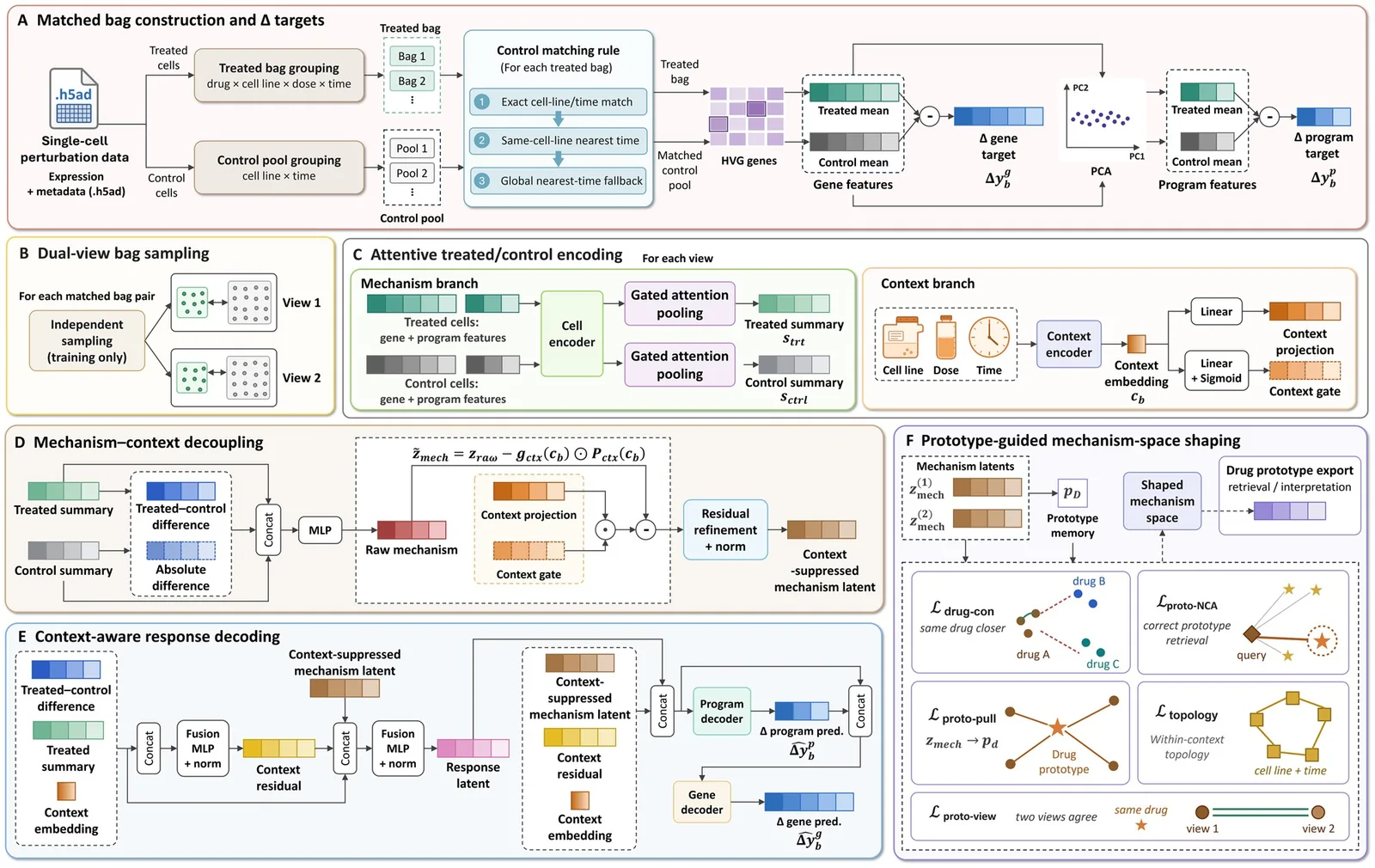
Overview of the DECANT framework. (a) Single-cell perturbation profiles are organized into matched treated-control bags, and bag-level gene and program perturbation targets, $\Delta y_{b}^{g}$ and $\Delta y_{b}^{p}$, are defined as treated-minus-control differences. (b) Two stochastic views are sampled from each matched bag pair during training. (c) Treated and control cells are encoded by a shared cell encoder and summarized by gated attention pooling, while cell line, dose and treatment time are encoded as context variables. (d) A raw perturbation representation is transformed into a context-suppressed mechanism latent by gated context subtraction and residual refinement. (e) A context-aware decoder predicts program- and gene-level perturbation responses. (f) Prototype-guided contrastive, retrieval, view-consistency, and topology-aware objectives shape the mechanism-aligned space for drug-prototype export, retrieval, and interpretation.

DECANT was developed to learn drug-centered, context-suppressed perturbation representations from single-cell chemical perturbation data while retaining the context-dependent information required for response prediction. As summarized in Fig. 1, the framework consists of four connected components: matched-bag input construction with dual-view sampling, attentive treated-control encoding, mechanism–context decoupling with response decoding, and prototype-guided mechanism-space shaping.

First, single-cell profiles are organized into matched treated-control bags, and two stochastic views are independently sampled from each matched bag pair during training (Fig. 1a and b). Each treated bag contains cells exposed to the same compound under a specific cell line, dose, and treatment time, and is paired with a matched control pool. Bag-level supervised responses are defined as treated-minus-control differences in gene and program space, denoted as $\Delta y_{b}^{g}$ and $\Delta y_{b}^{p}$.

Second, each stochastic view is encoded by a shared cell encoder followed by gated attention pooling (Fig. 1c). In parallel, cell line, dose, and treatment time are encoded as a context embedding $c_{b}$, from which a context projection and a context gate are derived.

Third, DECANT separates the perturbation representation into a context-suppressed, mechanism-aligned latent representation and context-dependent residual information (Fig. 1d and e). A raw perturbation representation is first computed from the attentive treated-control summaries. The gated context projection is then subtracted from this raw representation, followed by residual refinement and normalization. Importantly, this context-suppressed latent is not required to contain all response-relevant variation; residual context-dependent information is retained by the context-aware decoder to support accurate program- and gene-level response prediction.

Finally, during training, this forward process is optimized together with prototype-guided mechanism-space shaping objectives (Fig. 1f). These objectives encourage the learned representations to be stable across stochastic views, retrievable at the drug level and locally organized under controlled context comparisons.

### 2.2 Single-cell chemical perturbation dataset

We used the Sci-Plex 3 single-cell chemical perturbation dataset as the primary perturbational profiling resource (Srivatsan *et al.* 2020). The processed expression matrix contains single-cell transcriptomes annotated with compound identity, cell line, dose, treatment time, and control status. The dataset provides a multi-compound and multi-context perturbation setting, including three cellular backgrounds, A549, K562, and MCF7, and multiple dose conditions. This design enables evaluation of whether a model can recover drug-associated perturbational structure while controlling for variation introduced by cell line, dose, and treatment time.

The starting expression object contained 762 795 single-cell transcriptomes and 110 983 genes before downstream feature selection. We did not repeat raw sequencing-level processing, cell calling or mitochondrial-content filtering from sequencing files; instead, we used the curated single-cell expression matrix as the input to the computational pipeline. Downstream preprocessing retained cells with the metadata required to define perturbation conditions, namely drug identity, cell line, dose, treatment time, and control status. After feature selection, condition filtering, control matching, and minimum cell-count filtering, the final modeling dataset contained 2438 matched perturbation bags from 188 drugs.

### 2.3 Preprocessing and feature construction

Control status was determined from explicit control annotations when available. When explicit annotations were not available, control cells were inferred from control-like labels, including dimethyl sulfoxide, vehicle, control or untreated, and from zero-dose entries when dose information was present. Perturbed and control cells lacking the metadata required to define matched perturbation conditions were excluded from downstream bag construction.

Gene-level features were constructed in a fixed highly variable gene space. When the input expression object contained a precomputed feature-selection annotation, such as a highly variable or feature-selected flag, that annotation was used. Otherwise, genes were selected by variance computed on normalized log-expression values. For count-like matrices, expression values were normalized to a library size of 10 000 counts per cell and transformed by log(1+x) before feature scoring and matrix materialization (Wolf *et al.* 2018, Luecken and Theis 2019). Let $x_{i}^{g}\in R^{G}$ denote the selected gene-level feature vector of cell i, where G is the number of selected genes. For all reported experiments, *G* = 2000.

To capture coordinated transcriptional variation beyond individual genes, we constructed data-driven transcriptional program embeddings from the same selected gene matrix using principal component analysis (PCA; Abdi and Williams 2010). These programs act as unsupervised cellular pathway-like embeddings that summarize major gene co-expression axes, without relying on curated pathway labels. The PCA model was fitted using treated cells from the training split only, so that the program axes were estimated from perturbed transcriptional states while avoiding information leakage from validation or test drugs, and was then applied unchanged to all cells in the training, validation, and test splits. Let $W_{\text{prog}}\in R^{G\times K}$ denote the fixed PCA gene-to-program projection matrix, where K is the number of transcriptional programs, and let $\mu_{\text{prog}}\in R^{G}$ denote the PCA centering vector fitted on training treated cells. Under a column-vector convention, where $x_{i}^{g}\in R^{G}$ represents the gene expression profile and $x_{i}^{p}\in R^{K}$ represents the projected program profile, the program-level feature vector of cell i was computed as:


(1) $$x_{i}^{p}=W_{\text{prog}}^{⊤}\left(x_{i}^{g}-\mu_{\text{prog}}\right),\;x_{i}^{p}\in R^{K}.$$

In our downstream analyses, K=128. The same fitted program transformation was used for DECANT and all baselines, ensuring that program-level targets and metrics were defined in a common evaluation space (Huang *et al.* 2024, Guo *et al.* 2025, Huang *et al.* 2025).

### 2.4 Matched perturbation bags

The basic sample unit was a matched perturbation bag. A treated bag is defined as the set of single cells exposed to the same drug under the same cell line, dose, and treatment time. For bag b, we denote the treated-cell set by $B_{\text{trt},b}$, the matched control-cell set by $B_{\text{ctrl},b}$, and the drug identity by $D_{b}$. Control cells were grouped into control pools by cell line and treatment time. For each treated bag, a matched control pool was selected using an exact-first hierarchy: exact cell-line/time match, same-cell-line nearest-time match, and global nearest-time fallback when no same-cell-line control pool was available.

In the reported preprocessing configuration, treated bags were retained only when they contained at least 24 treated cells, and control pools were retained only when they contained at least 64 control cells. This bag formulation preserves single-cell resolution for attention-based modeling while defining deterministic bag-level supervised targets.

For each bag b, the treated and matched-control means in gene space were:


(2) $${\bar{x}}_{\text{trt},b}^{g}=\frac{1}{\left|B_{\text{trt},b}\right|}\sum_{i\in B_{\text{trt},b}}x_{i}^{g},\;{\bar{x}}_{\text{ctrl},b}^{g}=\frac{1}{\left|B_{\text{ctrl},b}\right|}\sum_{i\in B_{\text{ctrl},b}}x_{i}^{g}.$$

Analogously, the treated and matched-control means in program space were:


(3) $${\bar{x}}_{\text{trt},b}^{p}=\frac{1}{\left|B_{\text{trt},b}\right|}\sum_{i\in B_{\text{trt},b}}x_{i}^{p},\;{\bar{x}}_{\text{ctrl},b}^{p}=\frac{1}{\left|B_{\text{ctrl},b}\right|}\sum_{i\in B_{\text{ctrl},b}}x_{i}^{p}.$$

The supervised gene-level and program-level perturbation targets were defined as:


(4) $$\begin{array}{c} \Delta y_{b}^{g}={\bar{x}}_{\text{trt},b}^{g}-{\bar{x}}_{\text{ctrl},b}^{g},\\ \Delta y_{b}^{p}={\bar{x}}_{\text{trt},b}^{p}-{\bar{x}}_{\text{ctrl},b}^{p}=W_{\text{prog}}^{⊤}\Delta y_{b}^{g}. \end{array}$$

The quantities $\Delta y_{b}^{g}$ and $\Delta y_{b}^{p}$ are supervised bag-level targets. They are not provided to DECANT as direct input features. The model input consists of sampled treated cells, sampled matched-control cells, and context variables.

### 2.5 Fixed drug-level unseen-compound split and leakage control

All experiments used a fixed drug-level unseen-compound split. Drugs, rather than cells or bags, were assigned to training, validation and test sets, so that all bags from the same drug belonged to the same split. The final split contained 148 training drugs, 20 validation drugs, and 20 test drugs, corresponding to 1915 training bags, 256 validation bags, and 267 test bags. This setting evaluates generalization to drugs not observed during training rather than interpolation across new contexts of drugs already seen by the model.

This split should be interpreted as a fixed drug-level held-out-compound benchmark, not as a chemical-scaffold or mechanism-family split. Because partitioning was performed by drug identity, chemically related compounds and drugs belonging to mechanism families represented in the training set may also occur in the validation or test sets. Accordingly, the benchmark evaluates compound-level holdout generalization rather than scaffold-level extrapolation or generalization to previously unrepresented mechanism families. Test-drug bags were never used for model fitting, hyperparameter selection, early stopping or model selection. All fitted quantities used during model training and evaluation were estimated without using test-drug data. These included target-normalization statistics, PCA program construction, response-dominance weights used for $R_{w}^{2}$, and baseline-specific scaling parameters. The validation split was used only for hyperparameter tuning, early stopping, and model selection. The held-out test split was used only for final reporting.

### 2.6 Dual-view sampling of matched bags

This sampling procedure is designed to improve robustness to stochastic cell sampling within the same matched perturbation condition. During training, each matched treated-control bag pair was sampled twice to form two stochastic views. For view v∈{1,2}, DECANT sampled a treated subset $B_{\text{trt},b}^{\left(v\right)}\subseteq B_{\text{trt},b}$ and a matched-control subset $B_{\text{ctrl},b}^{\left(v\right)}\subseteq B_{\text{ctrl},b}$. The two views shared the same supervised targets $\Delta y_{b}^{g}$ and $\Delta y_{b}^{p}$, but were constructed from independently sampled cells. This design encourages the learned representation to remain stable under cell-level sampling variation within the same perturbation condition, consistent with the view-consistency principle used in contrastive representation learning (Chen *et al.* 2020, Khosla *et al.* 2020). During evaluation, deterministic bag-specific sampling was used to obtain reproducible predictions and embeddings.

### 2.7 Attentive treated-control encoding

This module summarizes heterogeneous treated and matched-control cell populations into compact bag-level representations while allowing informative cells to contribute more strongly through gated attention. For each sampled view, gene and program features were jointly encoded at the single-cell level. A shared cell encoder mapped each cell to a latent token:


(5) $$h_{i}^{\left(v\right)}=f_{\text{cell}}\left(x_{i}^{g},x_{i}^{p}\right).$$

Treated and matched-control tokens were summarized separately using gated attention pooling, following attention-based multiple-instance learning (Ilse *et al.* 2018). The unnormalized attention scores were computed from latent cell tokens using a parameterized gated-attention scoring network and then normalized by softmax within each treated or matched-control subset. The coefficients $\alpha_{\text{trt},i}^{\left(v\right)}$ and $\alpha_{\text{ctrl},i}^{\left(v\right)}$ are gated-attention weights satisfying:


(6) $$\sum_{i\in B_{\text{trt},b}^{\left(v\right)}}\alpha_{\text{trt},i}^{\left(v\right)}=1,\;\sum_{i\in B_{\text{ctrl},b}^{\left(v\right)}}\alpha_{\text{ctrl},i}^{\left(v\right)}=1.$$

The treated summary, matched-control summary and perturbation summary were defined as:


(7) $$\begin{array}{c} s_{\text{trt},b}^{\left(v\right)}=\sum_{i\in B_{\text{trt},b}^{\left(v\right)}}\alpha_{\text{trt},i}^{\left(v\right)}h_{i}^{\left(v\right)},\;s_{\text{ctrl},b}^{\left(v\right)}=\sum_{i\in B_{\text{ctrl},b}^{\left(v\right)}}\alpha_{\text{ctrl},i}^{\left(v\right)}h_{i}^{\left(v\right)},\\ \delta_{b}^{\left(v\right)}=s_{\text{trt},b}^{\left(v\right)}-s_{\text{ctrl},b}^{\left(v\right)},\;a_{b}^{\left(v\right)}=\left|s_{\text{trt},b}^{\left(v\right)}-s_{\text{ctrl},b}^{\left(v\right)}\right|,\\ u_{b}^{\left(v\right)}=\left[s_{\text{trt},b}^{\left(v\right)},s_{\text{ctrl},b}^{\left(v\right)},\delta_{b}^{\left(v\right)},a_{b}^{\left(v\right)}\right]. \end{array}$$

Here, [⋅,…,⋅] denotes feature concatenation along the channel dimension. The vector $\delta s_{b}^{\left(v\right)}$ is a latent difference between treated and matched-control summaries and is distinct from the supervised expression-space targets $\Delta y_{b}^{g}$ and $\Delta y_{b}^{p}$.

The context variables of bag b, including cell line, dose, and treatment time, were encoded as a context embedding:


(8) $$c_{b}=f_{\text{ctx}}(\ell_{b},d_{b},\tau_{b}),$$

where $\ell_{b}$, $d_{b}$, and $\tau_{b}$ denote the cell line, dose, and treatment time of bag b, respectively. The categorical cell-line variable $\ell_{b}$ was mapped to a learnable dense embedding, whereas the continuous dose and time variables $d_{b}$ and $\tau_{b}$ were represented as scaled numeric covariates and linearly projected before concatenation and input to $f_{\text{ctx}}$. The symbol $c_{b}$ is used exclusively for the context embedding. From $c_{b}$, DECANT derived a context projection $P_{\text{ctx}}(c_{b})$ and a context gate $g_{\text{ctx}}(c_{b})$. The context gate $g_{\text{ctx}}(c_{b})$ was implemented as a linear projection followed by a sigmoid activation, so that each gating value lies in [0,1].

### 2.8 Mechanism-context decoupling

This module is designed to suppress the component of the perturbation representation that can be attributed to measured context variables, while preserving context-dependent residual information for response decoding. Throughout this study, the term mechanism latent is used operationally to denote a drug-centered transcriptional perturbation representation designed to suppress variation attributable to measured context variables. Because DECANT is trained on transcriptional responses rather than direct target-engagement measurements or causal pathway interventions, this latent should be interpreted as a mechanism-aligned perturbation phenotype rather than as a direct representation of molecular mechanisms of drug action. The perturbation summary $u_{b}^{\left(v\right)}$ was mapped to a raw mechanism representation:


(9) $$z_{\text{raw},b}^{\left(v\right)}=f_{\text{raw}}\left(u_{b}^{\left(v\right)}\right).$$

To suppress context-associated variation in the mechanism branch, DECANT subtracted a gated context projection from the raw mechanism representation and then refined the context-subtracted latent:


(10) $$\begin{array}{c} {\tilde{z}}_{\text{mech},b}^{\left(v\right)}=z_{\text{raw},b}^{\left(v\right)}-g_{\text{ctx}}(c_{b})⊙P_{\text{ctx}}(c_{b}),\\ z_{\text{mech},b}^{\left(v\right)}=\text{Norm}\left[f_{\text{refine}}\left({\tilde{z}}_{\text{mech},b}^{\left(v\right)}\right)\right]. \end{array}$$

Here, ⊙ denotes element-wise multiplication, Norm[⋅] denotes layer normalization applied to the latent feature dimension, ${\tilde{z}}_{\text{mech},b}^{\left(v\right)}$ is the context-subtracted intermediate mechanism representation, and $z_{\text{mech},b}^{\left(v\right)}$ is the context-suppressed mechanism latent used for retrieval, prototype construction, cross-context stability analysis, and *post hoc* mechanism-aligned interpretation. Its biological alignment is subsequently evaluated using independent mechanism-family annotations and transcriptional consequence programs. It is not assumed to encode all context-dependent response variation; context-dependent residual information is retained for response decoding.

### 2.9 Context-aware response decoding

This module reconstructs gene- and program-level perturbation responses by combining the context-suppressed mechanism representation with residual context-dependent information needed for accurate prediction. Perturbation-response prediction was performed by a context-aware decoder. A context residual was derived from the latent treated-control difference, the treated summary, and the context embedding:


(11) $$r_{\text{ctx},b}^{\left(v\right)}=\text{Norm}\left[f_{\text{res}}\left(\delta s_{b}^{\left(v\right)},s_{\text{trt},b}^{\left(v\right)},c_{b}\right)\right].$$

A bag-level response latent was then formed by fusing the context-suppressed mechanism latent, context residual and context embedding:


(12) $$\begin{array}{c} h_{\text{bag},b}^{\left(v\right)}=f_{\text{bag}}\left(z_{\text{mech},b}^{\left(v\right)},r_{\text{ctx},b}^{\left(v\right)},c_{b}\right),\\ h_{\text{resp},b}^{\left(v\right)}=\left[h_{\text{bag},b}^{\left(v\right)},z_{\text{mech},b}^{\left(v\right)},r_{\text{ctx},b}^{\left(v\right)},c_{b}\right]. \end{array}$$

The program decoder first predicted the program-level perturbation response, and the gene decoder then predicted the gene-level perturbation response:


(13) $$\begin{array}{c} {\Delta y}_{b}^{p,(v)}=f_{\text{prog}}\left(h_{\text{resp},b}^{\left(v\right)}\right),\\ {\Delta y}_{b}^{g,(v)}=f_{\text{gene}}\left(h_{\text{resp},b}^{\left(v\right)},{\Delta y}_{b}^{p,(v)}\right). \end{array}$$

This design assigns distinct roles to the mechanism and response latents. The context-suppressed mechanism latent is optimized for drug-centered organization, retrieval, and interpretation, whereas the response latent preserves context-dependent variation required for accurate Δprogram and Δgene prediction.

Unless otherwise specified, the parameterized mapping functions in DECANT, including $f_{\text{cell}}$, $f_{\text{ctx}}$, $f_{\text{raw}}$, $f_{\text{refine}}$, $f_{\text{res}}$, $f_{\text{bag}}$, $f_{\text{prog}}$, and $f_{\text{gene}}$, were implemented as fully connected neural networks with Gaussian Error Linear Unit nonlinearities (Hendrycks and Gimpel 2016), dropout (Srivastava *et al.* 2014), and layer normalization (Ba *et al.* 2016) where appropriate. Residual refinement was used in the mechanism branch after context subtraction. The layer widths, latent dimensions, dropout rate, and optimization hyperparameters were fixed before the final multi-seed evaluation and were kept unchanged across the reported test runs.

### 2.10 Prototype-guided mechanism-space shaping

For brevity, we refer to the latent space defined by these context-suppressed representations as the mechanism space. This term denotes the model’s mechanism-aligned perturbation representation space and does not imply direct inference of molecular targets or causal mechanisms. This module shapes the context-suppressed mechanism representation into a drug-level geometry that supports retrieval, prototype construction, and biological interpretation. Because this geometry is explicitly optimized for drug-centered retrieval, its biological organization was assessed separately after training using independent mechanism-family annotations and downstream consequence programs. These external annotations were not used by the prototype-guided shaping objective. For each drug D, a prototype memory vector $p_{D}\in R^{H}$ was maintained as a drug-level anchor in the mechanism space, where H is the mechanism-latent dimension. Mechanism latents from the two stochastic views and the prototype memory jointly contributed to the shaping losses, producing a shaped mechanism space from which drug prototypes were exported after training for retrieval and interpretation.

The mechanism-space shaping objective contained five geometry-oriented terms. The drug contrastive loss, $L_{\text{drug-con}}$, encouraged mechanism latents from the same drug to be closer than those from different drugs (Khosla *et al.* 2020). Related supervised contrastive strategies have also been used in biomedical prediction tasks such as multi-functional therapeutic peptide prediction (Wang *et al.* 2024, Yan *et al.* 2024, Luo *et al.* 2025). The prototype pull loss, $L_{\text{proto-pull}}$, aligned bag-level mechanism latents with the corresponding drug prototype $p_{D}$.The prototype NCA loss, $L_{\text{proto-NCA}}$, made the correct drug prototype retrievable from the prototype memory (Goldberger *et al.* 2004). The prototype view loss, $L_{\text{proto-view}}$, enforced agreement between stochastic views at the drug-level mechanism representation (Chen *et al.* 2020, Khosla *et al.* 2020). The topology loss, $L_{\text{topology}}$, preserved local mechanism-space structure under matched cell-line/time conditions. This topology term acts on mechanism latents under controlled context conditions and does not make context embeddings themselves closer.

After training, exported drug prototypes were aggregated from learned mechanism embeddings and were distinct from both the online prototype memory and the fixed external LINCS/CMap teacher prototypes described in Section 2.11.

### 2.11 LINCS teacher prototypes and teacher-free control

This section defines the optional LINCS teacher component and the teacher-free control used to separate DECANT’s architecture from external teacher-derived transcriptional-response information. The teacher-free control corresponds to the w/o LINCS teacher ablation reported in Tables 5 and 6, available as supplementary data at *Bioinformatics* online.

External teacher prototypes were constructed from LINCS/CMap transcriptional-response signatures before DECANT optimization (Lamb *et al.* 2006, Subramanian *et al.* 2017). Compound identities were harmonized using canonical names and aliases, and teacher coverage followed a predefined matching procedure. For each covered compound or teacher group, available LINCS/CMap signatures were normalized and aggregated into a fixed external teacher prototype. These prototypes were fixed before DECANT training and were not updated using Sci-Plex training, validation or test bags.

The LINCS teacher component was used only as an optional refinement signal for training-drug representations. It did not define the primary prediction target, replace the matched treated-control response objective or provide information about held-out drugs. To avoid leakage under the fixed drug-level unseen-compound protocol, the teacher-guided refinement loss was applied only to training bags whose drugs had matched teacher coverage. Validation and test bags were never used to compute teacher loss, update teacher prototypes, fit model parameters, tune hyperparameters, determine early stopping, or select the final model. No teacher prototype lookup was performed for validation or test drugs during inference, prototype export, retrieval evaluation, or biological interpretation.

Because the LINCS teacher component introduces external transcriptional-response information, the full DECANT model should be interpreted as a teacher-guided variant. The w/o LINCS teacher ablation removes only the teacher-guided refinement term while retaining the same matched treated-control inputs, architecture, drug-level split, response-prediction losses, mechanism-context decoupling, retrieval-oriented shaping, optimization protocol, and evaluation metrics. This control tests whether DECANT’s main advantages remain without external teacher refinement.

Mechanism-family annotations, curated consequence modules, and downstream evaluation labels were used exclusively for *post hoc* biological evaluation and interpretation. None of these external biological information sources was used to construct teacher prototypes, train DECANT, tune hyperparameters, determine early stopping, select models, construct drug prototypes, or perform topology shaping.

### 2.12 Training objective and optimization

The training objective was organized according to the functional design of DECANT. The primary supervised term optimized bag-level gene and program response prediction, whereas the additional terms stabilized stochastic views, shaped the context-suppressed mechanism space, regularized context leakage, imposed dose-related consistency and incorporated optional LINCS/CMap teacher-guided refinement.

The total loss minimized during training was:


(14) $$L_{\text{total}}=L_{\text{resp}}+\lambda_{\text{view}}L_{\text{view}}+\lambda_{\text{shape}}(e)L_{\text{shape}}+\lambda_{\text{ctx}}L_{\text{deconf}}+\lambda_{\text{dose}}L_{\text{dose}}+\lambda_{T}(e)L_{\text{teacher}}.$$

The supervised response term was defined as:


(15) $$L_{\text{resp}}=\lambda_{g}L_{\text{gene}}+\lambda_{p}L_{\text{program}}.$$

Here, $L_{\mathrm{total}}$ denotes the total training loss and $L_{\text{resp}}$ denotes the supervised response-prediction loss. The terms $L_{\text{gene}}$ and $L_{\text{program}}$ supervise the bag-level gene and program perturbation targets, $\Delta y_{i}^{g}$ and $\Delta y_{i}^{p}$, defined in Section 2.4. The coefficients $\lambda_{g}$ and $\lambda_{p}$ weight the corresponding response losses, while the remaining coefficients balance the auxiliary view-consistency, mechanism-space shaping, deconfounding, dose-consistency, and teacher-guided terms.

The view-consistency term $L_{\text{view}}$ stabilized representations and predictions across the two stochastic views sampled from the same matched perturbation bag. The grouped shaping term $L_{\text{shape}}$ comprised the prototype-guided geometry objectives described in Section 2.10, including drug contrastive, prototype pull, prototype retrieval, prototype-view, and topology-aware terms. The deconfounding and dose terms regularized residual context alignment and dose-related response behavior, respectively. When external LINCS/CMap teacher prototypes were enabled, the teacher-guided term $L_{\text{teacher}}$ aligned eligible training-bag mechanism embeddings with the corresponding teacher prototypes, as described in Section 2.11. This term was removed in the w/o LINCS teacher ablation and was not computed for validation or test bags.

All balancing coefficients were fixed before final multi-seed evaluation and were not re-tuned on the test set. Epoch-dependent shaping and teacher-guided weights, $\lambda_{\text{shape}}(e)$and $\lambda_{T}(e)$, were introduced with warm-up or ramp schedules to stabilize early optimization. Model selection used a predefined validation-only composite score combining gene-level response prediction, program-level response prediction and retrieval quality. The held-out test split was used only for final reporting. Key implementation settings and fixed hyperparameters are summarized in Table 1, available as supplementary data at *Bioinformatics* online.

### 2.13 Fair baseline adaptation and comparison protocol

All baselines were evaluated under the same fixed drug-level unseen-compound matched-bag protocol as DECANT. The evaluation unit was a matched treated-control perturbation bag, and each method was required to produce a bag-level predicted gene response, ${\Delta y}_{b}^{g}$. DECANT directly predicted both gene- and program-level responses through its context-aware decoder. For baselines that produced only gene-level predictions, program-level responses were obtained using the same fixed PCA gene-to-program projection used to define the program targets. If a baseline natively predicted treated expression rather than a perturbation difference, its output was converted to a matched-control-relative gene response by subtracting the matched-control mean.

The comparison included strong simple baselines and adapted specialized perturbation models. The simple baselines were pseudo-bulk Δ + Ridge regression (PB Δ+ Ridge; Hoerl and Kennard 1970), pseudo-bulk Δ + partial least squares regression (PB Δ + PLS; Geladi and Kowalski 1986), pseudo-bulk Δ+ *k*-nearest neighbors regression (PB Δ+ kNN; Cover and Hart 1967), and mean-pooled treated/control features with a linear prediction head (Mean-pool + linear). The specialized baselines included scGen (Lotfollahi *et al.* 2019), BioLord (Piran *et al.* 2024), chemCPA (Hetzel *et al.* 2022), PRnet (Qi *et al.* 2024), and PerturbNet (Yu *et al.* 2025). Simple baselines directly predicted bag-level gene responses from matched pseudo-bulk or mean-pooled treated-control summaries, whereas specialized baselines were adapted to the matched-bag protocol by aggregating cell-level perturbed-expression predictions when needed. Structure-aware methods, including chemCPA, PRnet, and PerturbNet, retained their native molecular-structure inputs and were explicitly marked as structure-aware in the results.

Method-specific adaptations were limited to the interfaces required by the common matched-bag task. Where applicable, each method retained its native modeling assumptions, including scGen’s control-to-treated latent transformation, BioLord’s attribute-conditioned representation, chemCPA’s basal-state and perturbation composition, and the structure-aware drug representations used by PRnet and PerturbNet. Full model-specific adaptation, hyperparameter tuning and selected configurations are provided in Table 3, available as supplementary data at *Bioinformatics* online.

To avoid unfair information advantages, no baseline was given DECANT-specific components, including LINCS teacher distillation, prototype memory, drug-retrieval objectives, topology-aware regularization or context-deconfounding losses. Non-structure baselines were not given SMILES, molecular graphs or pretrained molecular encoders. Hyperparameter selection and early stopping were performed only on the validation split, and final performance was reported on the held-out test split across five independent random seeds. Because full DECANT includes LINCS teacher-guided refinement, benchmark results were interpreted together with the teacher-free ablation described above.

### 2.14 Response-prediction metrics

Let N denote the number of evaluated bags. For bag b, ${\Delta y}_{b}^{g}$, and ${\Delta y}_{b}^{p}$ denote predicted gene-level and program-level perturbation responses, and $\Delta y_{b}^{g}$ and $\Delta y_{b}^{p}$ denote the corresponding targets.

Gene-level Pearson correlation was computed as the mean row-wise Pearson correlation across evaluated bags:


(16) $$\rho_{g}=\frac{1}{N}\sum_{b=1}^{N}\text{corr}\left({\hat{\Delta y}}_{b}^{g},\Delta y_{b}^{g}\right).$$

Program-level Pearson correlation was computed analogously:


(17) $$\rho_{p}=\frac{1}{N}\sum_{b=1}^{N}\text{corr}\left({\hat{\Delta y}}_{b}^{p},\Delta y_{b}^{p}\right).$$

Here, corr(⋅,⋅) denotes Pearson correlation. Let $\Delta y_{b,j}^{g}$ denote the *j*-th scalar element of $\Delta y_{b}^{g}$ for gene j∈{1,…,G}. Gene-level root-mean-square error was:


(18) $${\text{RMSE}}_{g}=\sqrt{\frac{1}{NG}\sum_{b=1}^{N}\sum_{j=1}^{G}{\left({\Delta y}_{b,j}^{g}-\Delta y_{b,j}^{g}\right)}^{2}}.$$

Let $\Delta y_{b,q}^{p}$ denote the *q*-th scalar element of $\Delta y_{b}^{p}$ for program dimension q∈{1,…,K}. Program-level root-mean-square error was:


(19) $${\text{RMSE}}_{p}=\sqrt{\frac{1}{NK}\sum_{b=1}^{N}\sum_{q=1}^{K}{\left({\Delta y}_{b,q}^{p}-\Delta y_{b,q}^{p}\right)}^{2}}.$$

Response-dominance-weighted treated-expression coefficient of determination, denoted $R_{w}^{2}$, was reported as a secondary metric. For models predicting Δgene response, predicted treated expression was reconstructed as:


(20) $${\hat{y}}_{b}^{g,\text{trt}}={\bar{x}}_{\text{ctrl},b}^{g}+{\Delta y}_{b}^{g}.$$

The true treated expression was:


(21) $$y_{b}^{g,\text{trt}}={\bar{x}}_{\text{ctrl},b}^{g}+\Delta y_{b}^{g}.$$

Let $B_{\text{train}}$ denote the set of training bags. Gene weights were estimated from training data only. For gene j, the raw response-dominance weight was:


(22) $$\omega_{j}^{\text{raw}}=\frac{{\text{Var}}_{b\in B_{\text{train}}}\left(\Delta y_{b,j}^{g}\right)}{{\text{Var}}_{b\in B_{\text{train}}}\left(\Delta y_{b,j}^{g}\right)+\lambda_{R}{\text{Var}}_{b\in B_{\text{train}}}\left({\bar{x}}_{\text{ctrl},b,j}^{g}\right)+\epsilon_{R}}.$$

Here, ${\bar{x}}_{\text{ctrl},b,j}^{g}$ denotes the *j*-th gene of the matched-control mean for bag b, and all variances are computed over training bags $b\in B_{\text{train}}$. In our standard implementation, $\lambda_{R}=2.0$, $\epsilon_{R}=10^{-8}$, and $\gamma_{R}=2.0$. These constants were fixed before final evaluation and were applied identically to all methods. They were chosen to emphasize genes whose training-set perturbation variance was large relative to matched-control background variance, while suppressing genes dominated by control-state variation. Because $R_{w}^{2}$ was used only as a report-only secondary metric and was not used for training, early stopping, hyperparameter selection, or best-model selection, these constants do not affect model fitting or the primary benchmark ranking.

The final normalized weight was:


(23) $$\omega_{j}=\frac{\text{clip}{\left(\omega_{j}^{\text{raw}},10^{-8},1\right)}^{\gamma_{R}}}{G^{-1}\sum_{l=1}^{G}\text{clip}{\left(\omega_{l}^{\text{raw}},10^{-8},1\right)}^{\gamma_{R}}}.$$

Here, clip(x,a,b) clamps x to the interval [a,b], and l is a dummy gene index. The response-dominance-weighted treated-expression $R_{w}^{2}$ was then computed as:


(24) $$R_{w}^{2}=1-\frac{\sum_{b=1}^{N}\sum_{j=1}^{G}\omega_{j}{\left(y_{b,j}^{g,\text{trt}}-{\hat{y}}_{b,j}^{g,\text{trt}}\right)}^{2}}{\sum_{b=1}^{N}\sum_{j=1}^{G}\omega_{j}{\left(y_{b,j}^{g,\text{trt}}-{\bar{y}}_{j}^{g,\text{trt}}\right)}^{2}},$$

where ${\bar{y}}_{j}^{g,\text{trt}}$ is the mean true treated expression of gene j across evaluated bags. If the weighted total sum of squares was numerically degenerate, defined as less than or equal to $10^{-12}$, $R_{w}^{2}$ was set to zero. This metric was used only for reporting and was not used for model selection.

### 2.15 Retrieval and cross-context stability metrics

Drug-level retrieval was evaluated in the mechanism latent space using cosine similarity. For each query bag b, all other evaluated bags were ranked by cosine similarity to $z_{\text{mech},b}$, excluding the query bag itself. Retrieval recall at rank k, denoted R@k, and mean reciprocal rank (MRR) were defined as (Manning 2008):


(25) $$\begin{array}{c} R@k=\frac{1}{N}\sum_{b=1}^{N}1\left[\exists b^{\prime }\in {\mathrm{TopK}}_{-b}(b,k)\;\text{such}\;\text{that}\;D_{b^{\prime }}=D_{b}\right],\\ \text{MRR}=\frac{1}{N}\sum_{b=1}^{N}\frac{1}{r_{b}}. \end{array}$$

Here, $D_{b}$ is the drug identity of bag b,$r_{b}$ is the rank of the nearest non-self bag with the same drug identity as the query bag, 1[⋅] is the indicator function, and ${\mathrm{TopK}}_{-b}(b,k)$ denotes the set of the k nearest non-self bags to query bag b in the mechanism latent space. If no same-drug bag was retrieved, the reciprocal rank was set to zero. We report retrieval recall at rank 1 (R@1), retrieval recall at rank 5 (R@5), and MRR.

Cross-context cosine similarity was used to quantify representation stability across changes in cell line, dose or treatment time. For a representation vector $\xi_{b}$, the cosine similarity between two bags b and $b^{\prime }$ was:


(26) $$\text{cos}\;\left(\xi_{b},\xi_{b^{\prime }}\right)=\frac{\xi_{b}^{⊤}\xi_{b^{\prime }}}{||\xi_{b}{\mathrm{||}}_{2}\mathrm{||}\xi_{b^{\prime }}{\mathrm{||}}_{2}}.$$

Here, $\xi_{b}$ denotes the representation vector used for the corresponding cross-context analysis, and $\mathrm{||}\cdot {\mathrm{||}}_{2}$ denotes the Euclidean norm. Same-drug cross-context pairs were defined as bag pairs with the same drug identity and at least one differing context variable among cell line, dose, and treatment time. Different-drug same-context pairs were defined as bag pairs with different drug identities but matched context variables. This contrast was used to separate drug-associated stability from similarity induced by shared experimental context.

To assess residual measured-context contribution in the learned mechanism latent space, we performed two additional *post hoc* analyses using frozen bag-level DECANT mechanism embeddings from the same run as the main representation analysis. For variance partitioning, embeddings were L2-normalized and marginal $\eta_{F}^{2}$ for factor F was computed as:


(27) $$\eta_{F}^{2}=\frac{\sum_{g\in G_{F}}n_{g}{\left\|{\bar{z}}_{g}-\bar{z}\right\|}_{2}^{2}}{\sum_{b=1}^{N}{\left\|z_{b}-\bar{z}\right\|}_{2}^{2}}$$

Here, $z_{b}$ denotes the normalized mechanism embedding of bag b, N is the number of bags included in the analysis, $G_{F}$ denotes the set of levels of factor F, $n_{g}$ is the number of bags in level g, and ${\bar{z}}_{g}$ and $\bar{z}$ denote the corresponding group mean and overall mean embeddings, respectively. Drug identity, cell line, dose, and treatment time were evaluated separately, while joint measured context was defined as the composite label formed by cell line, dose, and treatment time. For context-direction sensitivity analysis, mechanism embeddings were standardized using statistics fitted on non-test bags, and auxiliary linear models were fitted to the measured context variables. Cell line and discretized dose and treatment-time labels were modeled using class-balanced logistic regression, whereas continuous dose and treatment time were modeled using ridge regression. The resulting coefficient vectors were stacked and orthogonalized by singular value decomposition to define a context-associated basis. This basis was removed by orthogonal projection, after which the residual embeddings were L2-normalized and the same pairwise statistics used in the main cross-context representation analysis were recomputed.

### 2.16 Matched-pair consequence analysis

For consequence-profile analyses, each drug was summarized by a fixed module-level consequence vector. We grouped predefined response-program features into nine broad perturbation-response axes: cell-cycle progression, proliferation/growth signaling, DNA-damage/p53 response, apoptosis, metabolic stress, hypoxia, proteostasis/unfolded protein response (UPR), protein secretion, and epithelial/cell-state programs. The source feature set comprised MSigDB Hallmark gene-set-derived program features (Liberzon *et al.* 2015) and selected PROGENy response program features (Schubert *et al.* 2018). These curated response-program features are distinct from the *K*-dimensional data-driven PCA program space used for DECANT input encoding, response prediction and benchmark evaluation. For each evaluated bag b, let $q_{b}$ denote the curated response-program vector, and let A denote the fixed program-to-module scoring matrix whose columns define the consequence modules. Module membership was specified in Table 4, available as supplementary data at *Bioinformatics* online. The matrix A was fixed before downstream evaluation and was not used for model training, hyperparameter selection or mechanism-space shaping. These modules were used as coarse-grained transcriptional consequence summaries rather than fine-grained evidence of causal mechanism of action (MoA) or direct target engagement, and may include proximal and secondary responses such as apoptosis, cell-cycle arrest, cellular stress, and toxicity-associated effects.

For drug D, let $B_{D}$ be the set of evaluated bags assigned to that drug. Under the column-vector convention for $q_{b}$, the drug-level consequence profile was defined as


(28) $$m_{D}=\frac{1}{\left|B_{D}\right|}\sum_{b\in B_{D}}A^{⊤}q_{b},\;m_{D}\in R^{M}.$$

Thus, $m_{D}$ represents the average module-level downstream consequence profile associated with drug D, rather than the drug prototype $p_{D}$ in the mechanism latent space. The curated response-program vector $q_{b}$ used for consequence profiling is distinct from the PCA-derived program response $\Delta y_{b}^{p}$, which was used only to quantify overall response magnitude for the matching analysis below.

Mechanism-family labels were assigned independently from ChEMBL-derived drug target and mechanism-of-action annotations (Zdrazil *et al.* 2024), after compound matching by canonical names and available chemical identifiers. Target annotations were manually harmonized into broader target-family and mechanism-family labels, and ambiguous or insufficiently annotated drugs were excluded from family-level comparisons. These labels were used only for external *post hoc* evaluation and biological interpretation, not for DECANT training, hyperparameter tuning, early stopping, model selection, prototype construction, or topology shaping. Different annotation granularities were used according to the analysis: Fig. 3b evaluated canonical families with at least five annotated compounds, Fig. 4 summarized broader inhibitor classes, and Fig. 5 examined pathway-level families with sufficient membership for response-matched comparisons.

Matched-pair comparisons were used to control for overall response magnitude. For drug D, the response magnitude was defined as the mean program-response norm across evaluated bags:


(29) $$s_{D}=\frac{1}{\left|B_{D}\right|}\sum_{b\in B_{D}}\mathrm{||}\Delta y_{b}^{p}{\mathrm{||}}_{2}.$$

Here, $s_{D}$ denotes the average program-level response magnitude of drug D; it was used only for response-magnitude matching. For two drugs $D_{1}$ and $D_{2}$, the response-magnitude difference was


(30) $$\delta_{\text{norm}}(D_{1},D_{2})=|s_{D_{1}}-s_{D_{2}}|.$$

Drug pairs were retained only when $\delta_{\text{norm}}$ satisfied the pre-specified matching criterion for the corresponding analysis. Directional similarity was then assessed by cosine similarity between consequence-profile vectors:


(31) $$\text{cos}(m_{D_{1}},m_{D_{2}})=\frac{m_{D_{1}}^{⊤}m_{D_{2}}}{\mathrm{||}m_{D_{1}}{\mathrm{||}}_{2}\mathrm{||}m_{D_{2}}{\mathrm{||}}_{2}}.$$

Mechanism-family labels were applied only after magnitude matching, so that the analysis tested family-specific consequence direction rather than differences in overall perturbation strength.

## 3 Results

### 3.1 DECANT shows the most favorable overall benchmark profile while preserving cross-scale consistency

**Figure 2:**
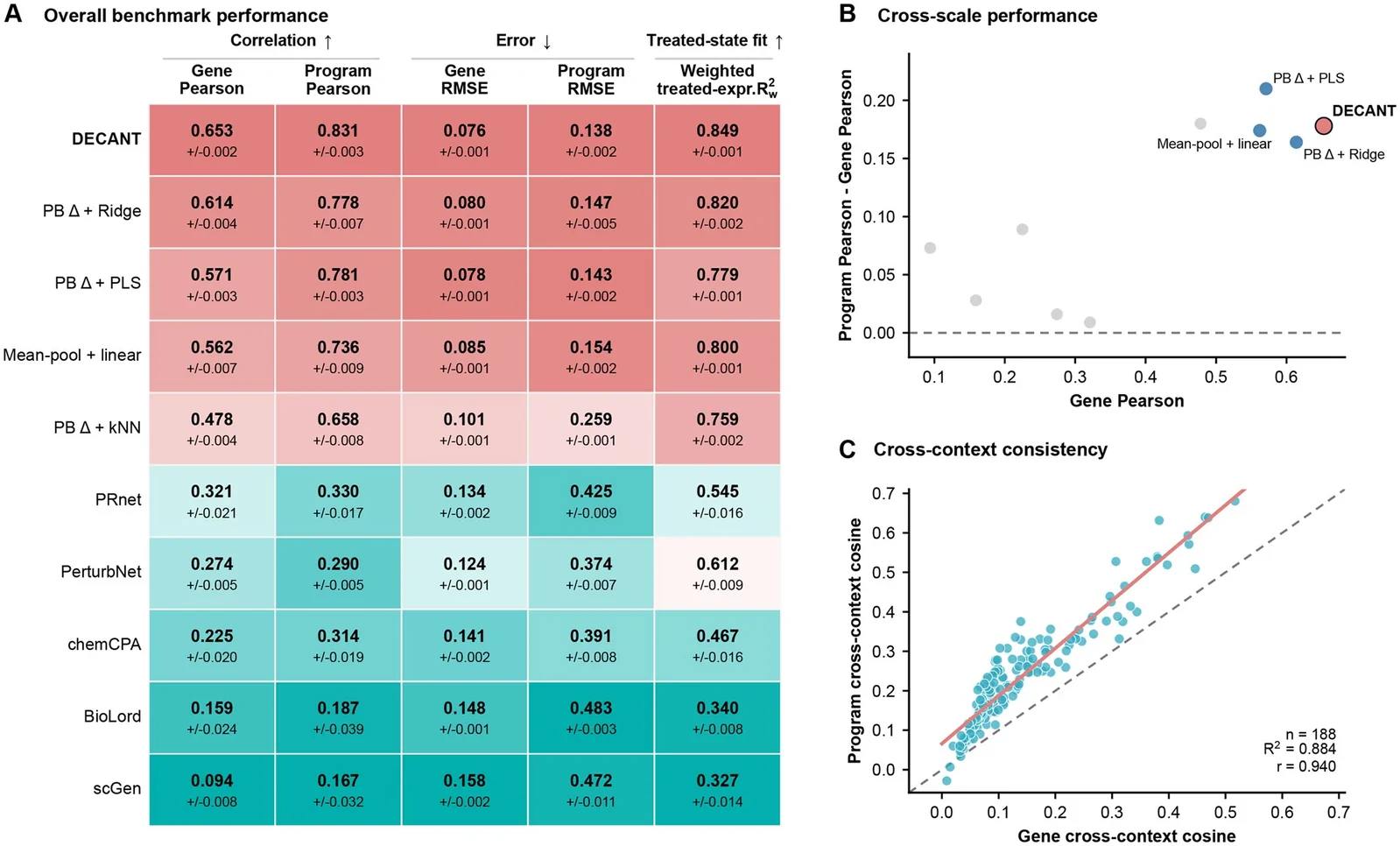
Benchmark performance and cross-scale consistency of DECANT. (a) Overall performance under the fixed drug-level unseen-compound protocol, comparing DECANT with adapted perturbation models and strong pseudo-bulk or mean-pooled baselines. Metrics include gene- and program-level Pearson correlation and RMSE; response-dominance-weighted treated-expression $R_{w}^{2}$ is reported as a secondary diagnostic. Values are mean ± SD across five independent seeds. (b) Program-level gain, defined as program-level Pearson minus gene-level Pearson, as a function of gene-level Pearson. (c) Relationship between gene-level and program-level cross-context cosine similarity across 188 drugs, with each point representing one drug.

To assess benchmark performance under fixed drug-level unseen-compound evaluation, we compared DECANT with two complementary baseline classes: adapted specialized perturbation models and strong bag-level or pseudo-bulk baselines (Fig. 2a). Across the four primary response-difference metrics, DECANT showed the most favorable overall profile, reaching a gene-level Pearson of 0.653 ± 0.002, a program-level Pearson of 0.831 ± 0.003, a gene-level RMSE of 0.076 ± 0.001, and a program-level RMSE of 0.138 ± 0.002 (mean ± SD across five independent seeds). The secondary response-dominance-weighted treated-expression metric, $R_{w}^{2}$, showed the same trend, with DECANT reaching 0.849 ± 0.001.

The adapted specialized models remained substantially lower under the common matched-bag protocol, spanning 0.094–0.321 in gene-level Pearson, 0.167–0.330 in program-level Pearson, and 0.327–0.612 in $R_{w}^{2}$. The strongest simple baselines formed the closest comparison group. PB Δ+ Ridge was the closest competitor in gene-level Pearson (0.614 ± 0.004) and $R_{w}^{2}$ (0.820 ± 0.002), whereas PB Δ+ PLS came closest in program-level Pearson (0.781 ± 0.003) and program-level RMSE (0.143 ± 0.002). Thus, DECANT’s predictive advantage over the strongest pseudo-bulk baselines was moderate but reproducible across independent initializations. Because the full model includes optional LINCS/CMap teacher-guided refinement, this benchmark is interpreted together with the teacher-free ablation reported below.

We next examined whether improved program-level agreement was achieved at the expense of gene-level fidelity. Program-level gain, defined as program-level Pearson minus gene-level Pearson, was plotted against gene-level Pearson across methods as a descriptive cross-scale diagnostic (Fig. 2b). DECANT occupied the most favorable region of this comparison, combining the highest gene-level Pearson (0.653) with a substantial program-level gain (0.178). This pattern indicates that DECANT did not trade gene-level reconstruction for higher-order program agreement, but instead maintained strong performance at both scales.

Finally, we asked whether this cross-scale pattern was mirrored by drug-level robustness across context shifts. Across 188 drugs, gene-level and program-level cross-context cosine similarity were tightly coupled (Fig. 2c), indicating that drugs stable across variation in cell line, dose, and time at the gene level also remained stable in program space. Taken together, DECANT combined the strongest response profile with coherent cross-scale stability, motivating subsequent analyses of retrieval, and biological organization.

### 3.2 Complementary components jointly support the predictive fidelity and retrieval structure of DECANT

To determine which components contributed to DECANT’s performance profile, we performed a structured ablation analysis in which each major component was removed while the remaining framework was kept unchanged (Tables 5 and 6, available as supplementary data at *Bioinformatics* online). The full model achieved the best joint prediction-retrieval profile across the four primary response-difference metrics, the secondary weighted treated-expression metric, and all three retrieval metrics.

The ablations revealed distinct component-specific roles. Removing mechanism-context decoupling produced the broadest deterioration across prediction and retrieval, supporting its role as the main signal-separation backbone. Removing retrieval shaping caused the largest collapse in drug-level retrieval while producing a more moderate decrease in response-prediction metrics, indicating that retrieval shaping primarily organizes local mechanism-space geometry.

The remaining components acted as stabilizing or refinement terms. The teacher-free variant remained competitive, indicating that LINCS teacher guidance refined but did not define DECANT’s performance advantage. Dual-view consistency and attention pooling supported robust bag-level representation learning, whereas topology-aware regularization mainly improved retrieval structure. Together, these ablations show that DECANT’s performance arises from complementary contributions across signal separation, retrieval shaping, and representation stabilization.

### 3.3 DECANT learns a context-robust drug-centered space enriched for independent mechanism-family annotations

**Figure 3:**
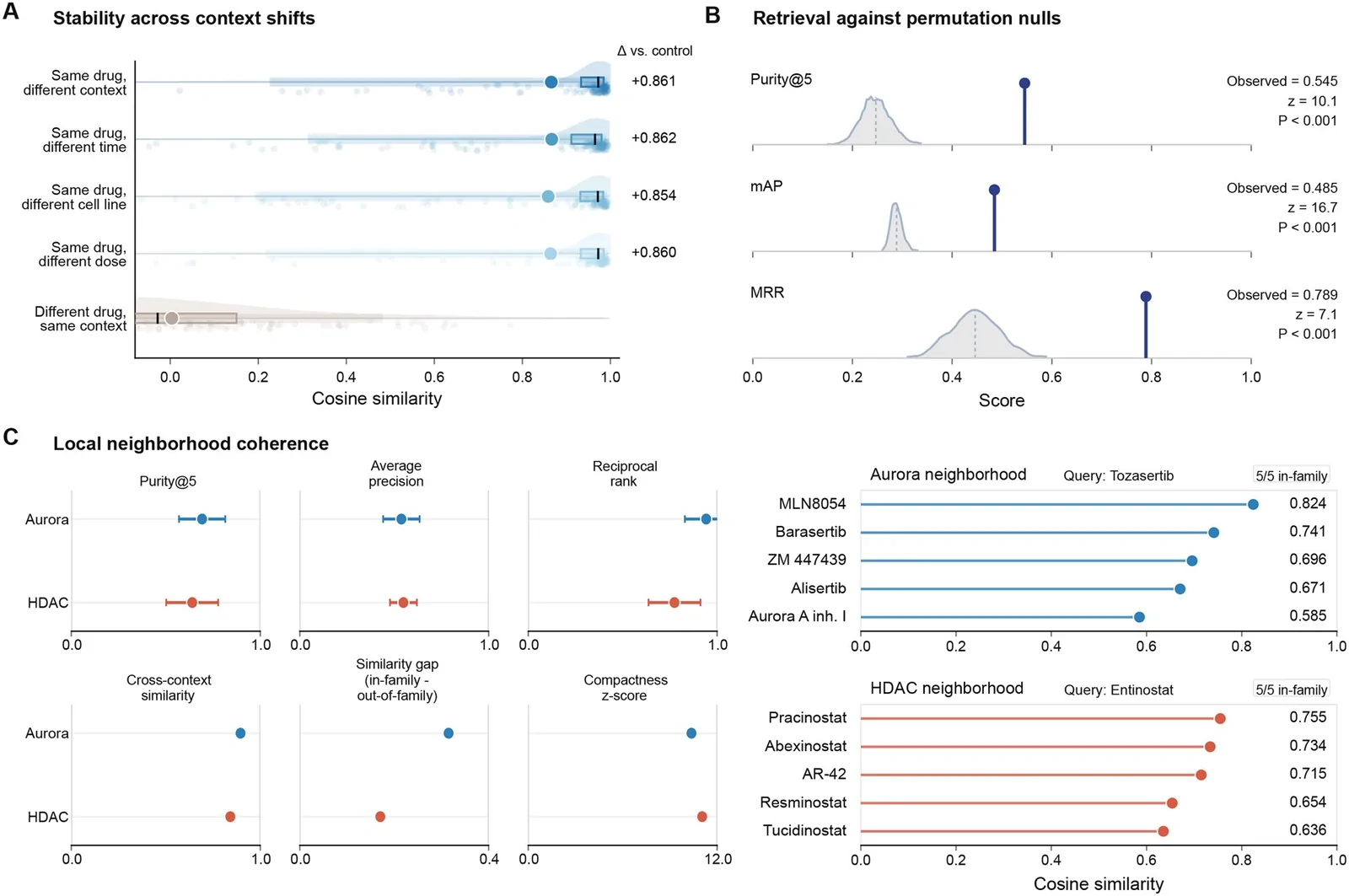
Context-robust embedding geometry and mechanism-family retrieval. (a) Pairwise cosine similarity of bag embeddings stratified by drug and context relationships, including same-drug pairs across dose, cell-line or time shifts, and different-drug pairs from the same context. Δ versus control denotes the difference in mean cosine similarity relative to the different-drug/same-context control stratum. (b) Retrieval enrichment of canonical mechanism families in the drug-prototype space, evaluated by Purity@5, mean average precision, and MRR. Permutation nulls were generated by shuffling mechanism-family labels while preserving family sizes. (c) Local neighborhood coherence for representative Aurora kinase and HDAC inhibitors, including family-level retrieval summaries and exemplar nearest-neighbor profiles.

Having established DECANT’s benchmark advantage, we next asked whether the learned representation was organized primarily by drug-associated perturbational signal rather than residual context identity. As a *post hoc* diagnostic, we embedded all 2438 processed bags without using mechanism-family labels for model fitting or selection. Same-drug relationships were strongly preserved across measured context shifts: relative to the different-drug/same-context control stratum, mean cosine similarity was higher by 0.860 across dose shifts, 0.854 across cell-line shifts and 0.862 across time shifts, with a pooled same-drug/different-context difference of 0.861 (Fig. 3a). By contrast, different-drug pairs from the same context were nearly orthogonal on average, with a mean cosine similarity of 0.004 across 214 708 pairs. Thus, shared context alone was insufficient to generate the high similarity observed for repeated measurements of the same drug.

To directly quantify residual measured-context contribution, we performed two complementary analyses on frozen DECANT mechanism embeddings. Variance partitioning showed that drug identity accounted for 87.4% of mechanism-latent variation, whereas joint measured context accounted for 1.16%; cell line, dose, and treatment time accounted for 0.224%, 0.068%, and 0.906%, respectively (Table 2, available as supplementary data at *Bioinformatics* online). We also removed context-associated directions estimated from non-test bags and recomputed the same pairwise statistics. Same-drug/different-context mean cosine similarity changed from 0.8656 to 0.8640, whereas different-drug/same-context similarity changed from 0.0044 to 0.0008. The same-drug minus same-context cosine gap remained essentially unchanged, from 0.8613 to 0.8632, and 99.55% of evaluated bags retained a positive gap. These results indicate that measured context contributes a limited fraction of the dominant latent-space variation.

We then evaluated whether the shaped drug-prototype geometry showed external biological alignment with independent mechanism-family annotations. Drug prototypes were constructed for all 188 compounds, and family-level retrieval was assessed on 66 evaluable drugs spanning five canonical families (Zdrazil *et al.* 2024). Under this evaluation, all three retrieval statistics lay well beyond their corresponding permutation nulls, with observed values of 0.545 for Purity@5, 0.485 for mean average precision, and 0.789 for MRR (all P<.001; Fig. 3b). These results do not imply perfect global segregation of all annotated mechanisms, but they show reproducible local enrichment of independent mechanism-family labels.

Local neighborhood analyses further supported this interpretation (Fig. 3c). Aurora kinase and histone deacetylase (HDAC) prototypes showed mean Purity@5 values of 0.692 and 0.640, respectively, with mean average precision values of 0.538 and 0.549. Both families also showed high cross-context similarity, positive in-family versus out-of-family similarity gaps and elevated compactness diagnostics. In exemplar neighborhoods, the top five nearest neighbors of Tozasertib were all Aurora kinase compounds, and the top five nearest neighbors of Entinostat were all HDAC compounds. Together, these analyses show that DECANT produces a context-robust, drug-centered perturbation-space geometry with stable same-drug embeddings and locally enriched mechanism-family neighborhoods.

### 3.4 DECANT neighborhoods align with interpretable downstream consequence programs

**Figure 4:**
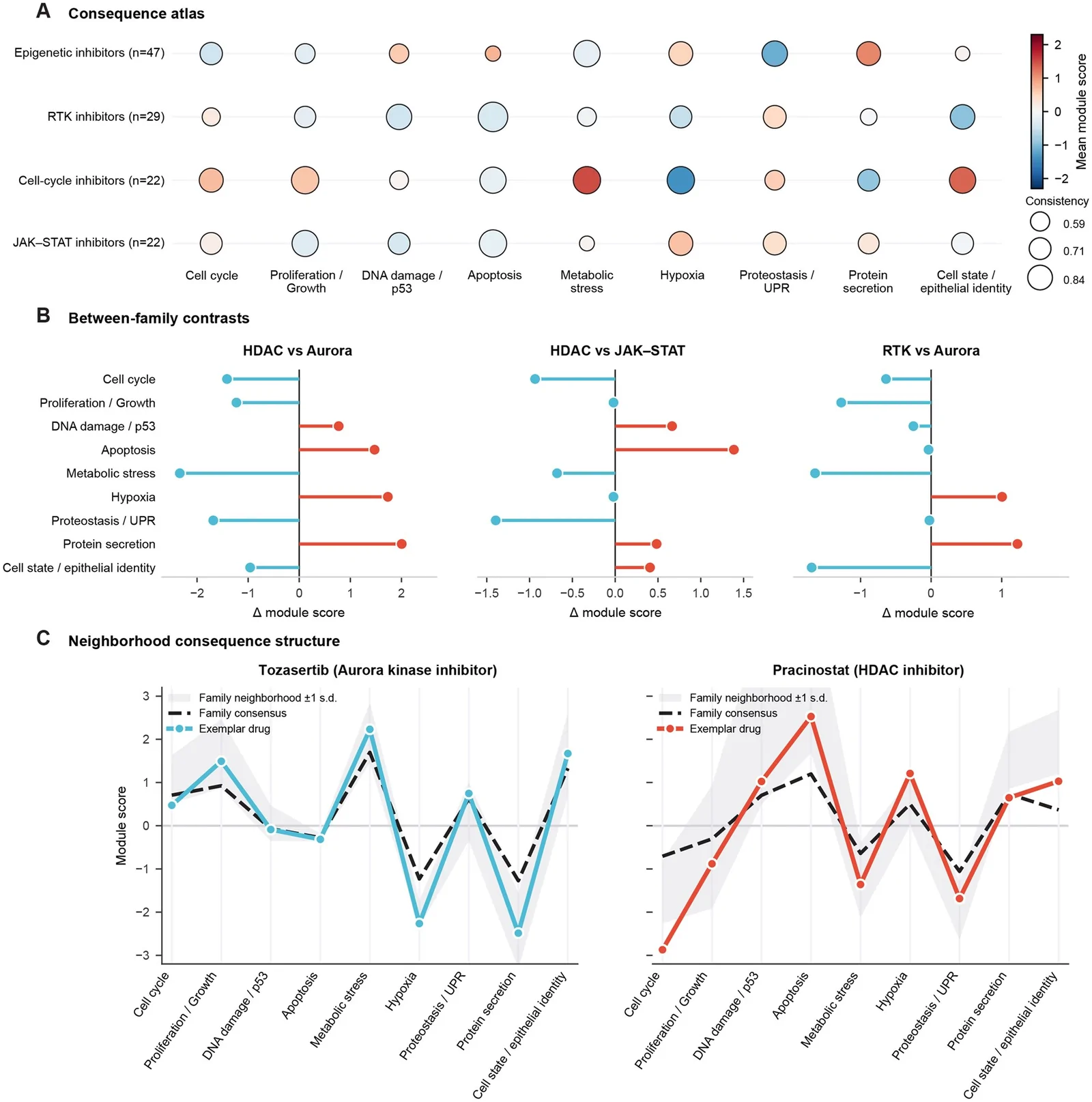
Downstream consequence programs associated with DECANT neighborhoods. (a) Module-level consequence atlas across major inhibitor groups. Color indicates the mean module score, and point size indicates module-wise within-family directional consistency. (b) Between-family consequence-program contrasts for selected family comparisons, shown as differences in mean module scores. (c) Consequence profiles of representative exemplar drugs compared with their family consensus and neighborhood dispersion. Module scores summarize coarse-grained transcriptional consequences and are not interpreted as direct measurements of causal MoA or target engagement.

Having shown that DECANT organizes drugs into context-robust and family-enriched neighborhoods, we next asked whether these neighborhoods also carried interpretable downstream consequence structure. We summarized module-level consequence scores across broad inhibitor classes, including epigenetic inhibitors, RTK inhibitors, cell-cycle inhibitors, and JAK–STAT inhibitors (Fig. 4a). These modules were constructed from predefined groups of MSigDB Hallmark gene-set-derived program features and selected PROGENy response program features (Liberzon *et al.* 2015, Schubert *et al.* 2018), as detailed in Table 4, available as supplementary data at *Bioinformatics* online. Accordingly, these analyses describe broad transcriptional consequence architecture associated with DECANT neighborhoods, rather than fine-grained validation of causal MoA or direct drug targets.

The resulting atlas showed that DECANT neighborhoods were associated with distinct consequence profiles rather than undifferentiated embedding clusters. Cell-cycle inhibitors displayed stronger cell-cycle/proliferation and metabolic-stress signals together with reduced hypoxia, whereas epigenetic inhibitors, including HDAC inhibitors, showed relatively stronger DNA damage/p53, apoptosis and protein-secretion signals and lower proteostasis/UPR. RTK, and JAK–STAT inhibitors occupied additional regions of this consequence landscape. Because the broad class-level map showed clear separation between epigenetic and cell-cycle regimes, we next examined target-family contrasts nested within these classes, focusing on HDAC inhibitors and Aurora kinase inhibitors.

These target-family comparisons showed that family separation reflected coordinated multiaxial consequence differences rather than a single dominant marker module (Fig. 4b). Relative to Aurora kinase inhibitors, HDAC inhibitors were shifted toward higher DNA damage/p53, apoptosis, hypoxia, and protein-secretion scores, together with lower cell-cycle/proliferation and metabolic-stress signals. A related but less extreme pattern was observed for HDAC versus JAK–STAT inhibitors, whereas RTK versus Aurora contrasts resolved along a different axis, with RTK neighborhoods comparatively enriched for hypoxia and protein secretion but reduced along growth- and cell-state-associated modules. Thus, family neighborhoods were not only geometrically coherent in representation space, but also interpretable in terms of downstream program architecture.

Finally, we examined representative exemplar drugs from the two highlighted target families: Tozasertib as an Aurora kinase inhibitor and Pracinostat as an HDAC inhibitor (Fig. 4c). Both exemplars broadly tracked their corresponding family consensus and remained directionally aligned with the neighborhood dispersion envelope across most modules. Tozasertib recapitulated the Aurora-associated pattern of elevated growth-related and metabolic-stress programs with low hypoxia and protein-secretion scores, whereas Pracinostat reproduced the HDAC-associated shift toward stronger DNA damage/p53 and apoptosis-related responses. Together, these analyses link local DECANT neighborhood geometry to coherent and interpretable transcriptional consequence programs.

### 3.5 Family-specific consequence structure persists after response-magnitude matching

**Figure 5:**
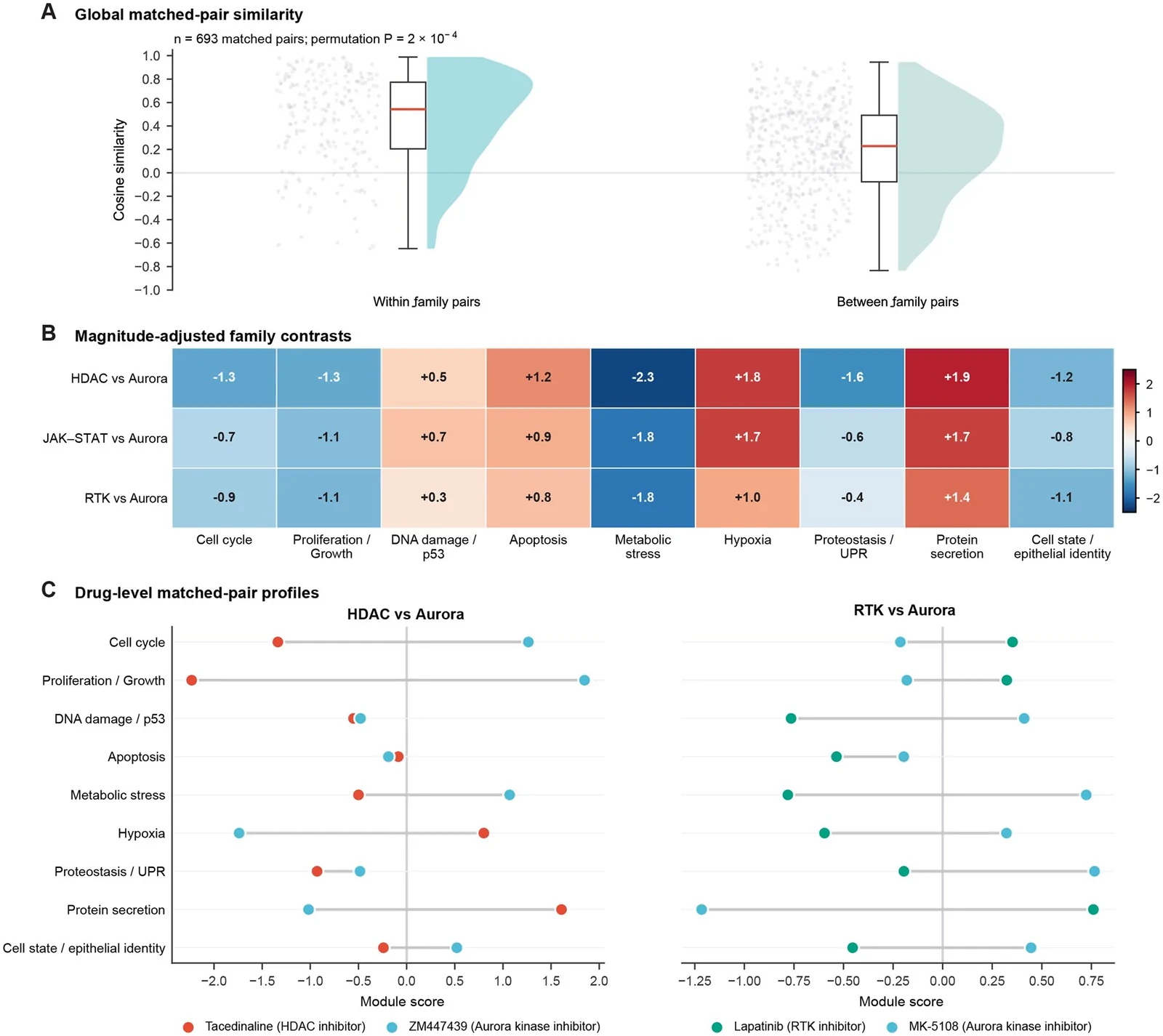
Family-specific consequence structure persists after response-magnitude matching. (a) Matched-pair similarity of module-level consequence profiles in the four-family subset. Drug pairs were retained when the absolute difference in mean program-response norm was ≤0.5, yielding 693 matched pairs. (b) Magnitude-adjusted family contrasts across nine consequence modules. Drug-level module scores were residualized with respect to mean response magnitude, and heatmap values show differences in family mean residuals. (c) Representative response-matched drug pairs showing module-level consequence differences despite similar overall response magnitude.

Unlike the pairwise bag-embedding analysis in Fig. 3a, which tested representation stability across context shifts, Fig. 5 asks whether drugs from the same mechanism family remain consequence-similar after controlling for overall response magnitude. We restricted this analysis to four pathway-level families with sufficient membership for matched-pair evaluation: histone deacetylation, Aurora kinase activity, JAK kinase activity, and RTK activity, defined from ChEMBL-derived target and mechanism annotations (Zdrazil *et al.* 2024). Each drug was summarized by the same nine-module consequence profile described above. This design shifts the focus from embedding proximity to whether mechanism families retain distinctive downstream consequence structure under a matched-response comparison.

At the global level, we computed cosine similarity between all drug pairs in this four-family subset and retained pairs with an absolute difference in mean program-response norm of at most 0.5, yielding 693 matched pairs. Within-family pairs remained substantially more similar than between-family pairs, with higher median cosine similarity (0.543 versus 0.228) and mean similarity (0.451 versus 0.181), and the family-coherence effect remained significant under permutation testing ($P=2\times 10^{-4}$; Fig. 5a). Because the same matching rule was applied to all candidate pairs, this comparison tests family structure under a controlled response-magnitude constraint rather than selecting pairs by outcome.

We next residualized each module score with respect to mean response magnitude and recomputed family contrasts on the adjusted profiles. Aurora kinase inhibitors served as the reference because they occupied one end of the proliferative and metabolic-stress-associated consequence spectrum in Fig. 4; this choice fixes contrast orientation but does not impose family separation. After adjustment, HDAC, JAK, and RTK families were all shifted downward relative to Aurora along cell-cycle, proliferation/growth and metabolic-stress axes, while shifting upward along apoptosis, hypoxia and protein-secretion programs (Fig. 5b). The HDAC-versus-Aurora comparison showed the strongest contrast, consistent with family-specific consequence architecture after removing global response-magnitude effects.

Finally, we examined two illustrative matched-response pairs: Tacedinaline versus ZM447439 for HDAC versus Aurora, and Lapatinib versus MK-5108 for RTK versus Aurora (Fig. 5c). In both cases, the paired drugs were closely matched in overall response magnitude yet separated in consequence direction. The HDAC–Aurora pair diverged most clearly along cell-cycle/proliferation, hypoxia and protein-secretion axes, while the RTK–Aurora pair preserved the same broad directional logic with more moderate module displacements. These exemplars provide drug-level illustrations of the global pattern in panels A and B, showing that family-specific consequence structure persists despite closely matched overall response magnitude.

## 4 Discussion

DECANT was developed to address a central challenge in single-cell drug perturbation modeling: transcriptional responses jointly reflect drug-associated perturbational effects and contextual variation from cell identity, dose, and treatment time. By combining matched treated-control bags, mechanism–context decoupling and prototype-guided representation shaping, DECANT learns a drug-centered perturbation representation that remains stable across measured context shifts while retaining response-prediction fidelity. Under the fixed drug-level unseen-compound benchmark, DECANT achieved the most favorable overall performance profile and supported cross-context stability analysis, drug-level retrieval, and *post hoc* biological interpretation. Strong pseudo-bulk and mean-pooled baselines remained competitive, indicating that DECANT’s predictive gains are consistent but moderate. Its main contribution therefore lies in coupling response prediction with a context-robust and biologically interpretable perturbation space, rather than in prediction accuracy alone.

The ablation analyses clarify the complementary roles of DECANT’s components. Mechanism–context decoupling provides the principal signal-separation backbone, whereas retrieval-oriented shaping contributes most directly to local drug-centered organization. Dual-view consistency, attention-based aggregation, and topology-aware regularization further stabilize the learned space, and the teacher-free ablation indicates that LINCS guidance acts primarily as a refinement signal rather than the sole source of DECANT’s advantage.

The learned representation also has a clear interpretive boundary. Because the geometry is explicitly shaped to support drug-centered retrieval, biological organization in this space is assessed externally rather than interpreted as purely emergent mechanism discovery. Independent mechanism-family annotations were enriched in local neighborhoods, and these neighborhoods were associated with coherent downstream consequence programs that persisted after controlling for overall response magnitude. These findings support biological alignment at the transcriptional-phenotype level, but they do not establish direct molecular mechanism, target engagement or causal pathway activity. Likewise, the Hallmark- and PROGENy-derived consequence modules should be interpreted as coarse-grained transcriptional outcomes that may include proximal responses as well as secondary effects such as apoptosis, cell-cycle arrest, cellular stress, and toxicity-associated responses.

Several limitations should guide interpretation of the current results. The fixed drug-level unseen-compound split evaluates generalization to held-out compounds within the matched-bag benchmark but is not a chemical-scaffold or mechanism-family split; related chemical structures and annotated families may therefore occur across training and held-out sets. The present results should accordingly be interpreted as compound-level holdout generalization rather than evidence of scaffold-level extrapolation or generalization to previously unrepresented mechanism families. Future work could extend DECANT to scaffold-aware or chemistry-stratified evaluation as larger and more chemically diverse perturbation atlases become available (Bemis and Murcko 1996). Prototype reliability may also decrease for drugs represented by few bags, low-cell-count conditions or weak perturbations. For multi-target compounds or drugs with strongly context-specific mechanism-associated responses, a single prototype may obscure distinct response modes across cell lines, doses or treatment times. Future extensions could therefore consider uncertainty-aware prototype estimation and context-stratified or multi-prototype representations. More complex applications may also require modeling additional context dimensions, including tissue microenvironment, multicellular composition and cell–cell communication (Armingol *et al.* 2021), as well as extension to multimodal perturbation profiles integrating transcriptomic and chromatin-accessibility measurements (Ma *et al.* 2020). Overall, DECANT provides a framework for learning context-robust, mechanism-aligned perturbation representations that support response prediction, drug-level retrieval and interpretable consequence analysis.

## Supplementary Material

btag662_Supplementary_Data

## Data Availability

The DECANT web server is publicly available at http://bliulab.net/DECANT. All source code and analysis scripts are available at https://github.com/bliulab/DECANT and archived on Zenodo at https://doi.org/10.5281/zenodo.21216567.
