# Supplementary material for: DECANT: decoupling mechanism from context in single-cell drug perturbation representation
Source: Bioinformatics. 2026 Sep 7;42(9):btag662. doi: 10.1093/bioinformatics/btag662 (PMC13587831; doi:10.1093/bioinformatics/btag662)
Supplement: Supplementary file 1 [file btag662_supplementary_data.docx]

**DECANT: Decoupling mechanism from context in single-cell drug perturbation representation**

**Ren Qi^1^, Wenjie Teng^1^, Xin Yang^1^, Yue Cheng^2,3^, Alexey K. Shaytan^4^, Bin Liu^1,2,5*^**

^1^ School of Computer Science and Technology, Beijing Institute of Technology, Beijing, 100081, China;

^2^ Zhongguancun Academy, Beijing, 100094, China;

^3^ School of Artificial Intelligence, Jilin University, Jilin, 130012, China;

^4^ Department of Biology, Lomonosov Moscow State University, Moscow, Russia;

^5^ SMBU-MSU-BIT Joint Laboratory on Bioinformatics and Engineering Biology, Shenzhen MSU-BIT University, Shenzhen, Guangdong 518172, China.

* Corresponding authors

**E-mail addresses of all authors**

RQ: rqi@bliulab.net

WJT: [wjteng@bliulab.net](mailto:wjteng@bliulab.net)

XY: yangxin@bliulab.net

YC: ycheng@bliulab.net

AKS: shaytan_ak@mail.bio.msu.ru

BL: [bliu@bliulab.net](mailto:bliu@bliulab.net)

**Mail addresses of the corresponding authors**

Bin Liu: Beijing Institute of Technology, No. 5, South Zhongguancun Street, Haidian District, Beijing 100081, China. Tel.: (+86) 010-68911310.

**Running Title:** DECANT for mechanism-aligned perturbation representations

**Table S1. Key DECANT implementation settings fixed before final evaluation.**

Response weights are reported as gene/program; view weights as latent/prediction consistency; shaping weights as drug-contrast/prototype-pull/prototype-view/prototype-NCA/topology; regularization weights as deconfounding/dose-ranking; teacher weights as distillation/neighbor consistency; validation selection weights as gene Pearson/program Pearson/R@5/MRR. All settings were fixed before final evaluation, and the held-out test split was used only for final reporting.

| **Component** | **Parameter or setting** | **Value** |
| --- | --- | --- |
| Input representation | Selected genes / PCA programs | 2,000 / 128 |
| Bag filtering | Minimum treated cells per bag / control cells per pool | 24 / 64 |
| Dual-view cell sampling | Training; evaluation | 112 treated + 224 control per view; 160 treated + 256 control per view |
| Latent dimensions | Cell token / mechanism / bag / context | 256 / 256 / 256 / 64 |
| Network width | Gene encoder / program encoder / attention pooling / decoder | 512 / 128 / 128 / 512 |
| Regularization | Activation / normalization / dropout | GELU / layer normalization / 0.10 |
| Prototype and similarity terms | Prototype momentum; contrast / topology / prototype-NCA temperatures | 0.92; 0.12 / 0.10 / 0.09 |
| Optimization | Optimizer / learning rate / weight decay / gradient clipping | AdamW / $1.6\times{10}^{-4}$/ $1\times{10}^{-4}$/ 1.0 |
| Training protocol | Epochs / steps per epoch / batch composition / patience | 120 / 80 / 10 drugs × 4 bags / 20 |
| Objective and selection | Loss weights; validation selection weights | response 0.95/0.85; view 0.12/0.18; shaping 0.26/0.24/0.14/0.24/0.16; regularization 0.06/0.05; teacher 0.06/0.015; selection 0.22/0.28/0.32/0.18 |

**Table S2. Residual measured-context contribution and context-direction sensitivity in the DECANT mechanism latent space.**

$\eta^{2}$ quantifies the fraction of mechanism-latent variation attributable to the indicated factor. Joint measured context denotes the composite context defined by the combination of cell line, dose and treatment time. Individual context-factor $\eta^{2}$ values were estimated separately and are therefore not intended to be additive. For the context-direction sensitivity analysis, context-associated directions were estimated from cell line, dose and treatment time using non-test bags and were removed post hoc from the frozen mechanism embeddings. Pairwise statistics were recomputed using the same same-drug/different-context and different-drug/same-context definitions as in the main representation analysis. Cosine gaps and changes were calculated from unrounded values. pp, percentage points.

| **Analysis** | **Quantity** | **Original mechanism latent space** | **After context-direction removal** | **Change** |
| --- | --- | --- | --- | --- |
| Variance partitioning | Drug identity $\eta^{2}$ | 87.4% | — | — |
| Variance partitioning | Joint measured context $\eta^{2}$ | 1.16% | — | — |
| Variance partitioning | Cell line $\eta^{2}$ | 0.224% | — | — |
| Variance partitioning | Dose $\eta^{2}$ | 0.068% | — | — |
| Variance partitioning | Treatment time $\eta^{2}$ | 0.906% | — | — |
| Context-direction sensitivity | Same-drug/different-context mean cosine similarity | 0.8656 | 0.8640 | −0.0016 |
| Context-direction sensitivity | Different-drug/same-context mean cosine similarity | 0.0044 | 0.0008 | −0.0036 |
| Context-direction sensitivity | Same-drug minus same-context cosine gap | 0.8613 | 0.8632 | +0.0020 |
| Context-direction sensitivity | Bags with a positive same-drug versus same-context gap | 99.55% | 99.55% | 0.00 pp |

**Table S3. Adaptation and hyperparameter tuning of specialized baselines under the common matched-bag benchmark.** All specialized baselines were evaluated using the same fixed drug-level split, matched-bag definitions, prediction targets and validation-based selection protocol. Hyperparameter tuning was performed separately for each method over its principal architecture, optimization and regularization parameters while preserving native modeling assumptions and input modalities. All hyperparameter and checkpoint selection relied exclusively on validation performance. The selected configuration of each method was then fixed for final multi-seed evaluation, and the held-out test set was not used for hyperparameter tuning or model selection.

(a) Adaptation to the common benchmark

| **Method** | **Native component retained** | **Task adaptation** | **Model selection** |
| --- | --- | --- | --- |
| scGen | Control-to-treated latent transformation | Cell-level predictions aggregated to predefined bags and converted to common gene/program responses | Validation only |
| BioLord | Attribute-conditioned latent representation | Predictions summarized under predefined bags in the common response space | Validation only |
| chemCPA | Composition of basal state, drug, dose and context | Matched-control bag representation used as basal input; outputs evaluated in common gene/program spaces | Validation only |
| PRnet | Structure-aware drug representation | Adapted to matched-bag inputs and common gene/program outputs while retaining structure information | Validation only |
| PerturbNet | Structure-conditioned perturbation representation | Adapted to matched-control and context-aware bag-level prediction while retaining structure conditioning | Validation only |

(b) Hyperparameter tuning and selected configurations

| **Method** | **Hyperparameter tuning and selected configuration** |
| --- | --- |
| scGen | Validation-based tuning of the principal model-capacity and regularization parameters. Selected: hidden dimension 256; latent dimension 64; dropout 0.20; batch size 32; max epochs 100; patience 25; program-level ridge $\alpha=1.0$. |
| BioLord | Validation-based tuning of the principal representation and attribute-regularization parameters. Selected: latent dimension 64; ordered/categorical attribute dimensions 16/16; attribute dropout 0.20; unknown-attribute noise and penalty 1.0; decoder lr $1\times{10}^{-4}$; attribute-network lr $3\times{10}^{-3}$; batch size 512; max epochs 40. |
| chemCPA | Validation-based tuning of the principal representation, optimization and adversarial-regularization parameters. Selected: latent dimension 256; hidden dimension 512; dropout 0.10; lr $2.5\times{10}^{-4}$; weight decay $1\times{10}^{-4}$; batch size 40; max epochs 120; warm-up 8 epochs; patience 20; drug/cell adversarial weights 0.02/0.01. |
| PRnet | Validation-based tuning of the principal architecture, optimization and regularization parameters. Selected: lr $1\times{10}^{-3}$; weight decay $1\times{10}^{-8}$; dropout 0.15; adaptor/encoder/decoder hidden size 128; batch size 40; max epochs 120; patience 20; response weighting $\lambda=2.0$. |
| PerturbNet | Validation-based tuning of the principal architecture, optimization and regularization parameters while retaining molecular-structure conditioning. Selected: Morgan fingerprint dimension 2048, radius 2; cell embedding 32; hidden dimension 512; dropout 0.25; lr $3\times{10}^{-4}$; weight decay $5\times{10}^{-5}$; batch size 64; max epochs 120; patience 20. |

**Table S4. Program composition of DECANT consequence modules.**

| Consequence module | Constituent MSigDB Hallmark gene sets | Additional PROGENy response programs |
| --- | --- | --- |
| Cell cycle | HALLMARK_E2F_TARGETS; HALLMARK_G2M_CHECKPOINT; HALLMARK_MITOTIC_SPINDLE | — |
| Proliferation/  growth | HALLMARK_MTORC1_SIGNALING; HALLMARK_PI3K_AKT_MTOR_SIGNALING; HALLMARK_MYC_TARGETS_V1; HALLMARK_KRAS_SIGNALING_UP | EGFR; PI3K; MAPK |
| DNA damage/p53 | HALLMARK_DNA_REPAIR; HALLMARK_P53_PATHWAY; HALLMARK_UV_RESPONSE_UP | p53 |
| Apoptosis | HALLMARK_APOPTOSIS | — |
| Metabolic stress | HALLMARK_XENOBIOTIC_METABOLISM; HALLMARK_FATTY_ACID_METABOLISM; HALLMARK_GLYCOLYSIS; HALLMARK_ADIPOGENESIS; HALLMARK_REACTIVE_OXYGEN_SPECIES_PATHWAY | — |
| Hypoxia | HALLMARK_HYPOXIA | — |
| Proteostasis/UPR | HALLMARK_UNFOLDED_PROTEIN_RESPONSE | — |
| Protein secretion | HALLMARK_PROTEIN_SECRETION | — |
| Cell state/epithelial identity | HALLMARK_ANDROGEN_RESPONSE; HALLMARK_ESTROGEN_RESPONSE_EARLY; HALLMARK_APICAL_SURFACE; HALLMARK_APICAL_JUNCTION; HALLMARK_EPITHELIAL_MESENCHYMAL_TRANSITION | — |

**Table S5. Ablation of response-prediction performance in DECANT under the fixed drug-level unseen-compound split.** Response-prediction performance of DECANT and six ablated variants was evaluated on the held-out drug-level test set. Metrics include four primary response-difference metrics: gene-level Pearson correlation, program-level Pearson correlation, gene-level root-mean-square error (RMSE) and program-level RMSE. Response-dominance-weighted treated-expression $R_{w}^{2}$ is reported as a secondary metric. Values are reported as mean ± s.d. across five independent seeds. Higher values indicate better performance for Pearson correlation and weighted treated-expression $R_{w}^{2}$, whereas lower values indicate better performance for RMSE.

| Model | Gene-level Pearson | Program-level Pearson | Gene-level RMSE | Program-level RMSE | Weighted treated-expression $R_{w}^{2}$ |
| --- | --- | --- | --- | --- | --- |
| w/o mechanism–context decoupling | 0.619 _± 0.006_ | 0.784 _± 0.004_ | 0.080 _± 0.001_ | 0.146 _± 0.003_ | 0.842 _±_ _0.002_ |
| w/o retrieval shaping | 0.626 _± 0.013_ | 0.789 _± 0.012_ | 0.078 _± 0.001_ | 0.144 _± 0.004_ | 0.830 _± 0.005_ |
| w/o LINCS teacher | 0.632 _± 0.006_ | 0.817 _± 0.006_ | 0.077 _± 0.001_ | 0.140 _± 0.003_ | 0.847 _± 0.003_ |
| w/o dual-view consistency | 0.621 _± 0.009_ | 0.788 _± 0.005_ | 0.078 _± 0.001_ | 0.142 _± 0.003_ | 0.844 _± 0.003_ |
| w/o attention pooling | 0.624 _± 0.006_ | 0.808 _± 0.003_ | 0.080 _± 0.001_ | 0.144 _± 0.003_ | 0.843 _± 0.002_ |
| w/o topology constraint | 0.637 _± 0.004_ | 0.812 _± 0.005_ | 0.078 _± 0.001_ | 0.141 _± 0.003_ | 0.848 _± 0.002_ |
| **DECANT** | **0.653 _± 0.002_** | **0.831 _± 0.003_** | **0.076 _± 0.001_** | **0.138 _± 0.002_** | **0.849 _± 0.001_** |

**Table S6. Ablation of drug-level retrieval performance in DECANT.** Retrieval performance of DECANT and six ablated variants was evaluated on the held-out drug-level test set. Metrics include retrieval recall at rank 1 (R@1), retrieval recall at rank 5 (R@5), and mean reciprocal rank (MRR). Values are mean ± s.d. across five independent seeds. Higher values indicate better retrieval performance.

| Model | R@1 | R@5 | MRR |
| --- | --- | --- | --- |
| w/o mechanism–context decoupling | 0.416 _± 0.020_ | 0.752 _± 0.017_ | 0.548 _± 0.013_ |
| w/o retrieval shaping | 0.256 _± 0.015_ | 0.598 _± 0.032_ | 0.430 _± 0.017_ |
| w/o LINCS teacher | 0.452 _± 0.023_ | 0.807 _± 0.012_ | 0.612 _± 0.017_ |
| w/o dual-view consistency | 0.470 _± 0.047_ | 0.786 _± 0.033_ | 0.575 _± 0.036_ |
| w/o attention pooling | 0.445 _± 0.025_ | 0.763 _± 0.023_ | 0.567 _± 0.016_ |
| w/o topology constraint | 0.439 _± 0.040_ | 0.792 _± 0.036_ | 0.600 _± 0.030_ |
| **DECANT** | **0.497 _± 0.015_** | **0.823 _± 0.020_** | **0.627 _± 0.017_** |
